# Codesigning a Culture-Centered Age-Friendly Community for Māori Kaumātua: Cultural Principles and Practices

**DOI:** 10.1093/geronb/gbac092

**Published:** 2022-07-07

**Authors:** Mary Louisa Simpson, John Oetzel, Yvonne Wilson, Sophie Nock, Kirsten Johnston, Rangimahora Reddy

**Affiliations:** Waikato Management School, University of Waikato, Hamilton, New Zealand; Waikato Management School, University of Waikato, Hamilton, New Zealand; Te Rūnanga o Kirikiriroa, Hamilton, New Zealand; Faculty of Māori and Indigenous Studies, University of Waikato, Hamilton, New Zealand; Rauawaawa Kaumātua Charitable Trust, Hamilton, New Zealand; Rauawaawa Kaumātua Charitable Trust, Hamilton, New Zealand

**Keywords:** Housing, Cultural factors, Qualitative methods

## Abstract

**Objectives:**

This study examined a Māori (Indigenous people of Aotearoa New Zealand) age-friendly housing development. Two Māori community groups worked with multiple stakeholders to codesign a culture-centered, kaumātua (older adults) urban housing community. The purpose was to identify codesign and culture-centered principles in the development.

**Methods:**

Kaupapa Māori (Māori-centered) and participatory research methodologies guided the culture-centered research design. Data collection included 27 interviews with 19 residents and 12 organizational stakeholders; three focus groups with residents’ families, service providers, and nonresident *kaumātua* (*n* = 16); and project documents. Data analysis used the framework method.

**Results:**

Three codesign process themes emerged: (a) Kaumātua-centered vision; (b) realizing the vision; and (c) living the shared vision.

**Discussion:**

Accounting for cultural practices in codesigning age-friendly and culture-centered housing for and with Indigenous older adults helps meet their cultural, social, health, and economic needs. The research offers a practical pathway to developing age-friendly housing environments for Māori *kaumātua*, their communities, wider society, and other Indigenous people.

Age-friendly environments are purposed with optimizing people’s functioning as they age and strategically shift the design of communities for aging populations (World Health Organization, 2007, 2018) to include housing, transport, respect and social inclusion, civic participation and employment, outdoor spaces, communication and information, and community support and health (Plouffe & Kalache, 2010; Wahl & Gerstorf, 2020). Earlier research, however, recognized the significance of age-friendly environments and associated housing needs (Cantor, 1975; Lawton & Simon, 1968). For example, housing and environments need to fit people’s changing functionality as they age (Lawton & Simon, 1968; Wahl & Gerstorf, 2020). Furthermore, a life space supports healthy aging and aging-in-place by offering services and transportation, walkable and inviting social spaces, and neighborhoods that sustain social capital (Cantor, 1975). Finally, functionality and life space needs for housing exist within multicontextual environments, including physical, economic, and social elements (Wahl & Gerstorf, 2020).

The current study examined housing for Māori *kaumātua* (Indigenous older adults) within Aotearoa New Zealand. Māori comprise about 17% of the population, of which about 7% are aged 65 years or older (Statistics New Zealand, 2020). Economic, physical, and social elements comprise a large context of housing and life spaces and consequences for *kaumātua*. Economic factors shape where *kaumātua* live (Cram & Munro, 2020; Goodyear, 2017); for example, the current housing crisis of rising rents and housing costs result from limited housing supply and increased demand (Lees, 2019). Also, Māori homeownership has fallen, with less than half owning their home compared with two thirds of the general population (Goodyear, 2017). Low homeownership affects *kaumātua* particularly badly because the universal (retirement) remuneration was established at a time of greater homeownership across all ethnic groups. Furthermore, Aotearoa has limited protection in rental jurisdiction (e.g., short leases, short eviction notices; Cram & Munro, 2020). These economic factors result in rising rates of Māori living in poor, temporary, or overcrowded housing (Cram & Munro, 2020; Goodyear, 2017; James et al., 2022) and overrepresentation of Māori in social and temporary housing (Baker et al., 2016). The flow-on effect of poor housing is profound poor health and well-being for Māori (Baker et al., 2016; Pledger et al., 2019).

Physical functioning also shapes housing for *kaumātua*. Many older people desire to age within age-friendly life spaces (Chyr et al., 2020; James & Saville-Smith, 2018). However, several trends negatively affect aging-in-place and life spaces internationally (Campbell-Enns et al., 2020; Chyr et al., 2020). For example, the housing cost burden (over 30% of income on housing) increases the chances of moving to a nursing home (Jenkins et al., 2020). Furthermore, scarcity of age-friendly housing as residents age and their needs change also inhibits aging-in-place (Frochen & Pynoos, 2017; van Hoof et al., 2021; Luciano et al., 2020). In a longitudinal study of octogenarians, social isolation and older age were central predictors of Māori moving to long-term residential care (Holdaway et al., 2021).

Cultural factors are vital to social connectedness, housing, and life-space elements. For example, older people may prefer to socialize with others from similar cultural backgrounds and share taken-for-granted social customs and knowledge (Morgan et al., 2021). Likewise, the philosophical grounding of Māori *tikanga* (cultural practices) and *te ao Māori* (Māori worldview) are essential for *kaumātua* (Durie, 2003). *Kaumātua* have important roles and are revered in Māori communities (Taskforce on Whānau-Centered Initiatives, 2010). Being carriers of culture with a desire to live within a cultural life-space as Māori are likely reasons for *kaumātua* not preferring long-term residential care (Holdaway et al., 2021). However, despite the reverence and wish for cultural connectivity, a growing segment experiences social isolation due to moves away from rural settlements and *whānau* (extended family) moving away from *kaumātua* for work (Wright St Clair et al., 2017).

The prominent housing inequities of Māori are widely seen as unfair, preventable, and correctable (Jones et al., 2019) and part of a larger historical and social context affected by colonization, structural racism, and unjust distribution of social determinants (Pledger et al., 2019). Many *iwi* (tribes) and Māori social service providers have sought their own solutions to housing inequities and the needs of *kaumātua*. For example, in the current study, two Māori community organizations worked collaboratively with *kaumātua*, builders and financiers to develop a housing community, or *papakāinga* (traditional Māori housing/community complex), and provide wrap-around services to meet urban *kaumātua* needs. Rather than wait for the government to address such needs, these organizations developed a village and subsequent toolkit for other Māori organizations to develop age-friendly vibrant and thriving *kaumātua* communities.

We adopted an age-friendly frame to explore the development and outcomes of the above *kaumatua* housing community. We took the view that “age-friendly” aspects of housing and community are those features that help to optimize people’s lives as they age (World Health Organization, 2018). Such aspects include respect and social inclusion, community and health support, civic and social participation (World Health Organization), with a focus on living environment, physical spaces, social characteristics, and participatory design activities (van Hoof et al., 2021). From a *te ao Māori* perspective, a housing village is part of a larger community that ideally should support *kaumātua* well-being and help them connect culturally and socially as well as support them to lead age well. Thus, the article examines the development of the *kaumātua* village within a broader context of age-friendliness.

The theoretical and methodological He Pikinga Waiora (HPW) Implementation Framework that guided this study uses participatory codesign approaches, centers Māori knowledge (*mātauranga Māori*), and emphasizes self-determination (Oetzel et al., 2017). HPW argues that interventions and research with Indigenous communities should be grounded in Indigenous knowledge and worldviews and include four dimensions: culture centeredness, community engagement, systems thinking, and integrated knowledge translation. Culture centeredness suggests that practical project and system transformation happens when Indigenous cultural perspectives define problems and solutions (Dutta, 2007). Community engagement is a collaborative process between groups directly affected by a particular issue and other groups working with those most affected (Wallerstein et al., 2018) and one that is advocated for developing age-friendly cities and communities (Blakey & Clews, 2020; van Hoof et al., 2021). Systems thinking helps address the complexity of local contexts and various levels and determinants of housing problems (Frerichs et al., 2016). Finally, integrated knowledge translation emphasizes codesign and coproduction with end users in developing and implementing an intervention (e.g., age-friendly village) to transfer knowledge and enhance sustainability (Grimshaw et al., 2012).

The purpose here is to examine the codesign process using the HPW framework to identify key features of the housing community and to create transferable knowledge for other community organizations. Specifically, the following research question framed the study: What codesign and culture-centered principles and practices helped develop the *kaumātua* village?

## Method

In 2017–2018, we used a culture-centered research design to examine a built and lived-in age-friendly Māori *kaumātua* housing community in Aotearoa. In 2012–2014, Te Rūnanga o Kirikiriroa (n.d.; Te Rūnanga) developed the *kaumātua* community in partnership with the Rauawaawa Kaumātua Charitable Trust (n.d.; Rauawaawa) that provides wrap-around services (i.e., supervision, care, and support) for residents. To meet changing *kaumatua* needs, *kaumātua* and Rauawaawa were involved in the design, planning, and project team.

The HPW framework provided the methodology, particularly *Kaupapa Māori* (Māori-centered) and participatory research processes. *Kaupapa Māori* is a research philosophy grounded in *tikanga* as “for-Māori-by-Māori” that seeks to transform research and promote Māori ways of being by normalizing Māori worldviews, language, culture, and autonomy in research and valuing participants’ voices (Kennedy & Cram, 2010; Smith, 2012). Participatory research involves partners throughout and privileges community self-determination, community-identified issues, different ways of knowing, community–researcher collaboration, and codesigned research (Wallerstein et al., 2018).

Our research team reflected a long-term partnership of Māori community researchers from Te Rūnanga and Rauawaawa, who coded the project, and one Māori and two *Pākēhā* (European New Zealander) University researchers. Te Rūnanga focuses on issues relating to equality and meeting the multifaceted needs of urban Māori, including housing. Rauawaawa provides “wrap around” support, programs, and services to enhance the quality and enjoyment of life for *kaumātua* aged 55 years and older. The Board Advisory Group comprised Trustees (all *kaumātua*) from both agencies, and the Expert Advisory Group comprised professionals in housing, social services, and research. We sought guidance from these groups at quarterly meetings.

### Participants

Forty-seven participants (29 females; 18 males) took part, with most (90%) identifying as Māori. These included 35 *kaumātua*, *whānau*, and service providers and 19 *kaumātua* residents aged 59–95 years (12 females; 7 males), along with 6 residents’ *whānau* members (female), 4 nonresident *kaumātua* (2 females; 2 males), and 6 Rauawaawa staff providing services (female). The remaining 12 (4 females; 8 males; aged mid-30s to 80 years) were organizational stakeholders (i.e., Rauawaawa and Te Rūnanga visionaries; external planners, project managers, construction team members; housing sector agencies, and funders).

### Data Collection

Interview questions sought to elicit stakeholders’ experience during the visioning, planning, and building processes and residents’ experiences of living in the village. Focus group questions focused on support for residents and participants’ experiences in caring for and interacting with residents.

Questions for Rauawaawa, Te Rūnanga, and external organizational participants focused on roles in the housing project, goals, benefits and barriers to achieving goals, relationships with others involved, and success factors. Questions for *kaumātua* residents related to their housing and community experience; health and financial status before and after moving to the village; circumstances that prompted moving; benefits and challenges of living there; and community involvement. Finally, focus group questions focused on what participants knew about the original vision for the kaumātua community and the extent of its achievement.

The Te Rūnanga property manager personally invited residents to participate, and a Māori researcher interviewed participants in their homes. The researcher took food to share and offered participants the choice of beginning and ending with *karakia* (prayers). Next, the researchers invited organizational participants by phone or email, conducting interviews at an agreed location. Finally, participants received a voucher to recognize their contribution.

Interviews were audio-recorded and transcribed, and interviewees were offered their transcripts for review and approval. During the focus groups, a researcher took detailed notes and summarized the discussion points for participants. Finally, project documents (e.g., plans and meeting minutes) were collected.

### Data Analysis

We used a framework analysis method (Gale et al., 2013) comprising three philosophical domains of the Māori world (Marsden, 1992) that provided a coding lens for inductive analysis of the transcripts. *Te Korekore* focused on the potential, vision, and initial planning of the project. *Te Pō* focused on the project “becoming” and considered relationships among stakeholders and the building process. *Te Ao Mārama* emphasized being; residents’ lived experiences and support within the village. Within each, we looked for expressions of Māori values and principles. Four researchers (three Māori, one Pākehā) independently coded selected interview transcripts, compared codes, and then coded the remaining transcripts independently. Next the initial themes and subthemes were identified through a comparative process and finalized.

## Results

Three themes addressed the research question, “What codesign and culture-centered principles and practices helped develop the *kaumātua* village?”: (a) Kaumātua-centered visioning; (b) realizing the vision; and (3) living the shared vision. To protect identities, we used nondemographic coding to cite data sources: O# for KCC/Te Rūnanga, project consultants, housing sector, and funding representatives; KR# for *kaumātua* residents; FG for *Whānau* members’ focus group; FGW for workers’ focus group; and MM# for documents (e.g., meetings, proposals).

### Theme 1: Kaumātua-Centered Vision

Theme 1 concerned the vision developed through codesign and the vision’s potential to address *kaumātua* needs associated with housing, culture, and later life. The vision (*moemoeā*) incorporated two aspects: a culture-centered *papakāinga* for *kaumātua* and a codesign process involving *kaumātua*.

The first aspect, culture-centered urban *papakāinga*, manifested Rauawaawa and Te Rūnanga’s response to the needs of urban Māori *kaumātua*. The original vision responded to the increasing homelessness of urban *kaumātua*: “[some] had been sleeping on a couch for years” (O1). Te Rūnanga identified *kaumātua* among many Māori needing accommodation: “it was a mess actually—we decided we would get into housing” (O2).

The vision for urban *kaumātua* was more than housing; it was a pathway to building a culture-centered age-friendly housing community with, and for, *kaumātua.* Values of cultural connectedness and relationships, and that “it takes a village to look after each other” (O2), underpinned the vision. The vision was for dwellings “to be built and arranged so that nobody would ever get sick without someone else in the village knowing” (O2). The vision was for a built environment that fostered and supported relationships (*whanaungatanga*) and mutual care (*manaakitanga*).

Rauawaawa and Te Rūnanga envisaged the *kaumātua* village as an urban “*papakāinga*” (O2). Traditionally, *papakāinga* are settlements on Māori ancestral lands where *whānau* live and look to return after living away. In contrast, the proposed urban *kaumātua* village comprised purchased land owned by Te Rūnanga with support services managed by Rauawaawa. The land acquisition and management arrangement raised two questions: (a) why was this village just for *kaumātua* and (b) if it was only for *kaumātua*, could the village be a *papakāinga* (O1, O2, O12)? Rauawaawa and Te Rūnanga identified *kaumātua* who had lived in the city for many years (often for work reasons); were unable or unwilling to return to their traditional lands; and, for some, were disconnected from their *whānau*. Te Rūnanga and Rauawaawa envisaged “a *papakāinga*” as a “holistic supported, deliberate community” (O12) that was a purposeful, coordinated, and collective response to the housing and social needs of urban *kaumātua*.

In traditional *papakāinga*, support is primarily communal and *whānau* connected. The urban *papakāinga* vision offered structured support from Te Rūnanga and Rauawaawa in addition to *kaumātua* care of each other. Several participants (O5, O8, O9, O10, O11, O12) and the original visionaries (O1, O2, O3) commented on the significance of promoting care and support. For example, Rauawaawa and Te Rūnanga “made sure it was as much about the wrap-around support for *kaumātua* as it was about the houses” (O12). The vision was “a place that was culturally safe for our kaumātua” founded on “the partnership between Te Rūnanga and Rauawaawa” (O1). The inclusion of wrap-around services and housing within the urban *papakāinga* created a package of social and environmental support to meet the changing needs of *kaumātua*.

In the second aspect, the codesign process involving *kaumātua*, the principles of collaboration and *kotahitanga* (unity) were central. *Kaumātua* were involved in codesigning the age-friendly community by first identifying the different problems they experienced. Next, *kaumātua* were asked “if they were in charge (…), what would their housing look like” (O2), and how would the design support their lives culturally. One housing sector representative commented that Rauawaawa and Te Rūnanga asked “*kaumātua* what they want; how do they want to live” (O12; also, O3, O8, O10). Thus, the “design and placement of the houses and community spaces [were] driven by engagement with the (…) community and [how it] is going to operate” (O12). Overall, the vision for a *kaumātua* village manifested Māori values of care and hospitality (*manaakitanga*) and connectedness (*whanaungatanga*) in an age-friendly urban community.

### Theme 2: Realizing the Vision

Theme 2 captured actions and *Māori* values that facilitated realizing the vision of the *kaumātua* community. Such values as *whakahihikotanga* (inspiration, motivation), *whanaungatanga*, *kaitiakitanga* (stewardship) and *tautokotanga* (support) were evident in the project.

The first aspect highlighted connections between *Māori* values, relationships (*whanaungatanga*), and motivations (*whakahihikotanga*) to support the project practically. Many organizational participants had long-standing relationships with Rauawaawa and Te Rūnanga members and also with each other: “we had been in this community for many years, (…) gone away to work [elsewhere], and (…) come back, so we had relationships that were 20-years-plus” (O8). This relational history meant “having a really good understanding of the community itself (…) and having respect for *whānau*, *hapū* [kinship group] and *iwi*—that was so important and just trying to keep it as Māori as possible” (O8). Thus, being Māori with experience in the housing sector made it easy to embrace the vision.

Other housing sector participants recognized the vision’s value and the potential of new relationships to realize that vision. For example, one participant saw their role was to “*awhi* [embrace] (…) that vision” (O10); another alluded to respect for the leadership behind the vision: “they were pretty smart” (O11). These and other participants (e.g., O8, O12) recognized that they could support the vision within their respective housing sector agencies.

Many project partners sought to contribute because the project offered affordable housing for older Māori. One participant said, “to provide quality accommodation for *kaumātua*” (O4) was the key to his involvement. Another was motivated because it was “a nice little project, there is a need for it, and you needed to have some models” (O11). A third saw it as “one of the best investments” in social housing for *kaumātua* at that time (O12). Finally, the vendor was motivated to sell the land of the “community housing” (O6) focus.

The second aspect concerned two closely connected actions: stewardship and support. Stewardship (*kaitiakitanga*) centered on managing the day-to-day team communication and overcoming practical hurdles during the project. Support (*tautokotanga*) was direct and indirect and also often associated with practical problems. Such support included accessing financial resources, navigating the resource consent application (formal council approval for the project), and dealing with neighborhood resistance.

Identifying stakeholders with technical knowledge of regulatory and financial systems or with experience in health and social services and working with *whānau* and *kaumātua* demonstrated stewardship. Some participants also recognized these efforts as leadership within Rauawaawa and Te Rūnanga: “they were pretty smart in (…) in having [named person] (…) to drive the people (…) across the whole thing” (O11) and “bring everything together” (O3). Stewardship was especially evident in the relationship-building approach: for example, “We wanted them to get to know us and us to get to know them, so it was much easier for us to go and talk to them about issues” (O2). Fundamental to sharing knowledge and skills was the aspiration to build collaborative and communicative relationships that would endure beyond the project.

Several participants mentioned team communication within project management. For example, they noted face-to-face “around the table” (O1), “weekly site meetings” (O9), and the documents detailed planning, progress, and problem-solving (MM1). In addition, the project records referred to resident consultation, project updates, budget and finance, meetings with the builder and planner, and “setting up a committee to collate criteria for housing *kaumātua*” (MM1). Participants’ comments also indicated collaboration: everyone “worked together” (O3), “had good communication” (O4), and “respect[ed] others’ views” (O2).

One critical issue was accessing project finance. Housing sector supporters who understood the regulations and policies helped with this. For instance, to gain local-body consent to build in an area “predominantly zoned Residential” (MM2), the *kaumātua* village was described as a “managed care facility” (MM2). Banks, however, were reluctant to lend to Te Rūnanga because of revenue uncertainty (O1). Instead, funders from various housing sector and government agencies (O1, O2, O7, O10, O11, O12) helped develop a funding model that included government agency support. One participant explained the contractual and relational significance of one source: “we [funder] wanted to support (…) and partner with Te Rūnanga to build affordable and social houses [so] the grants came with conditions about the relationship” (O12). In this respect, the vision for *kaumātua* supported living, facilitated access to funding from government agencies with similar aspirations for social housing.

The leadership behind the vision of a *kaumātua* urban *papakāinga* became shared through long-standing and new relationships. The vision was endorsed by those able to provide tangible support, resulting in greater potential for realizing the vision. Participants expressed commitment to *kaumātua*, recognized the need for affordable housing, and saw the potential of the community project to support *kaumātua*.

### Theme 3: Living the Shared Vision

Data collection comprised three components: (a) 27 semistructured organizational stakeholder and resident (individual and joint) interviews; (b) three focus groups: one each with family members, service providers, and nonresidents; and (c) project documents (e.g., meeting notes, building plans, planning documents). The University of Waikato granted ethical approval (12/09/18).

Theme 3 described how resident *kaumātua* and partner organizations perceived and enacted the vision once the was village occupied. The aspects of this theme centered on how *kaumātua* experienced the original vision of *papakāinga* in caring for each other, receiving organized support, sharing their experience to benefit others, and how Rauawaawa and Te Rūnanga enacted the post-tenancy partnership with *kaumātua.*

The first aspect of enacting or living the vision concerned the built environment. The *kaumātua* village comprised two adjacent sites of six and eight one- and two-bedroom insulated homes based on universal design principles for older people’s mobility and functional needs. The homes were positioned around the perimeter facing inward to shared common areas that supported residents’ interaction and *kaumātua*-supporting-*kaumātua* (e.g., for socializing). Also, the “garden (…) was low maintenance, (...) and manageable [for *kaumātua*]” (O2). The built environment helped create an environmental age-friendly setting that facilitates connections between residents.

The second aspect of living the original vision of a *papakāinga* was evident in *kaumātua* living on shared land, with care and support from each other and a sense of belonging. Two illustrative comments from *kaumātua* were as follows: “we’re always visiting (…) it’s a good way of life” (KR1) and “it’s like my second family” (KR11). Others noted the company and relational bonds (KR1, KR5) and the Māori culture centeredness of the village, referring to it as “*marae*-like” (Māori community meeting place; KR1; KR7; FG; FGW). The social and environmental dimensions of a *papakāinga* fostered social interaction among residents.

The papakāinga embedded care and hospitality (*manaakitanga*) and a sense of safety and security*. Kaumātua* stressed the importance of give-and-take in their community: “we help each other” (KR1) and “if I fall [spiritually/emotionally] I know my mates here [will] come over and say, ‘Come on, we’re going out’” (KR11). Finally, comments from *kaumātua* show they valued Rauawaawa services: for instance, “you’ve got the support here” (KR6) and “the nurses are very good, they transport us to our medical appointments” (KR7). The wrap-around services were central to care and support.

In addition, *kaumātua* expressed self-determination and independence in their everyday lives. One *kaumātua* echoed the sentiments of other residents when they commented on village living, “I will still hav[e] my independence, and (…) stand on my own two feet” (KR11). Residents initiated activities and helped create their village by self-funding social events and buying extra equipment for themselves (KR1; KR12).

The third aspect of enacting the vision revealed two parts to the post-tenancy village management. First, Te Rūnanga developed a relationship plan that identified how to involve residents and other stakeholders “in as many aspects of the organization as practicable” (MM3). The Te Rūnanga offered residents training to build capacity to participate in village affairs; developed policies and transparent processes to support early identification of issues; and established regular, formal, and informal communication opportunities (MM3). Together these aimed to foster *kaumātua*–organization partnership.

Second, Rauawaawa and Te Rūnanga worked with government agencies, which resulted in Te Rūnanga “leasing the properties back to [organization], and we managed it like any other tenancy (…) including the tenancy application. We would do the interviews but allowed Rauawaawa to decide” (O8). In this way, Rauawaawa was able to identify and offer a home to *kaumātua* with the greatest needs. The funding contract incorporated high-level cooperation: “Rauawaawa would provide the wrap-around services (…) and Te Rūnanga would build the houses” and “the rent (…) and maintenance side of things were guaranteed [by organization]” (O10). It was a relationship that worked well (O8, O10, O11, 012) and a partnership that ensured a culturally safe age-friendly community with secure tenancy.

The shared vision was an extension of the lived vision; participants thought it was important to share their experience to benefit other *kaumātua.* For example, one resident showed how the vision for the *kaumātua* community applied to them: “I wouldn’t leave this place. I said to my kids … ‘I’m here to stay’” (KR11). Other *kaumātua* residents (e.g., KR1, KR6, KR7), *whānau* (FG), and workers (FGW) reported similar sentiments. Residents shared their positive experiences of village living with their *whānau*, friends, and workers.

At an organizational level, the lived vision was shared “as a nationally recognized project (...) we’ve had many groups come and visit—a wonderful outcome” (O2). In addition, a government housing publication (Ministry of Business, Innovation and Employment, 2014) and a toolkit (Reddy et al., 2019) currently in use by different communities featured the *kaumātua* village’s lived vision. Sharing lived experiences reinforces important culturally centered principles within the codesign, building, and tenancy management phases.

Collectively, the three themes represented various phases of creating a *kaumātua papakāinga* within *te ao Māori* and showed vital facilitating processes in codesigning a *kaumātua* community. Table 1 summarizes these processes as objectives, principles, and practical questions.

**Table 1.** Facilitating Processes for Codesigning a Māori *Kaumātua* Village/*papakāinga*

Objectives to achieve	Practices, principles, values to apply	Questions to address
1. Create a clear shared vision and aspiration for *kaumātua* housing (drawn from Theme 1)	• Work within *Te Ao Māori* • Care passionately about *kaumātua* • Commit to the vision and working together • Co-create a solution-focused vision with *kaumātua*	• What is the agreed, shared vision? • What does it take for the vision to be maintained and nurtured?
2. Create and maintain long-term, high-trust, collaborative relationships (drawn from Themes 1 and 2)	• Establish long-term relationships with key stakeholders • Connect with like-minded people across the sector • Identify/connect with strategic allies in regulatory systems • Co-create collaborative relationships/effective communication • Coordinate/lead stakeholder relationships	• Who can best help achieve the vision? • What new/existing high-trust relationships support achieving the vision? How to create and maintain such relationships?
3. Use systems/processes that benefit the housing project (drawn from Themes 1, 2)	• Work with strategic allies in regulatory systems • Develop monitoring systems for project budget and progress • Schedule regular project team meetings	• How to use regulatory standards/ rules to meet *kaumātua* housing needs? • Who can best help with these?
4. Create and maintain a *kaumātua* community (drawn from Themes 1 and 3)	• Build relationships with each other •* Kaumātua* support each other •* Kaumātua* support create a sense of belonging • Promote peace of mind for *kaumātua* in terms of both community and tenancy	• How does the built environment support *kaumātua* to *manaaki* (care for) and *tautoko* (support) each other?
5.* KaumātuaMana motuhake* (drawn from Theme 3)	•* Kaumātua* enact self-determination and independence •* Kaumātua* organize their own funds for village events, etc. •* Kaumātua* drive village activities •* Kaumātua* work in partnership with property management	• How do *kaumātua* create and maintain the village for *kaumātua*? • How do the built environment and management systems support *kaumātua mana motuhake*?
6. Wrap-around support for *kaumātua* with multiple service providers (drawn from Themes 1 and 3)	• Provide care and support • Maintain housing and property • Involve *kaumātua* in monitoring maintenance • Maintain high-trust relationships among providers	• What are *kaumātua* health and social needs? • How does organizational processes and systems support those needs?
7. Culture-centered, *kaumātua* community and housing (drawn from Themes 1 and 3)	• Create marae-like setting and culture • Embed Māori values in all aspects • Codesign communal spaces to support village activities and whanau/visitors (e.g., *tangi*/funeral) • Enable *whānau* (family) to stay for periods	• What are *kaumātua* cultural needs? • How does the built environment and systems support those needs? • How are Māori values embedded in community, design, systems, etc.?
8. Partner with *kaumātua* in codesign to meets their (changing) needs (drawn from Themes 1, 2, and 3)	• Adopt age-friendly principles in housing, grounds, etc. • Engage *kaumātua* at critical stages (e.g. design, review) • Co-create facilities for providers to care and stay over • Develop systems to monitor residents’ changing needs	• What are current and potential *kaumātua* needs? • How does the built environment and systems support those needs?
9. Share experiences, learning, and knowledge (drawn from Theme 3)	• Expose other providers to potential opportunities • Facilitate *kaumātua* involvement in *kaumātua* housing • Evaluate project outcomes and strengths • Develop a resource or toolkit for others	• How to connect with others to help groups develop creative options to meet the housing needs of *kaumātua*?

## Discussion

This study aimed to identify essential codesign and culture-centered principles in a *kaumātua* housing community development. These principles may benefit Māori and other Indigenous community groups in codesigning age-friendly communities. The overarching success factors encompassed (a) the culture-centered approach to age-friendly principles, (b) community agency, and (c) practicalities in construction and postconstructions phases.

### Culture-Centered Age-Friendly Principles

The findings highlighted important culturally grounded processes that centered the traditional Māori philosophy of *papakāinga* in developing and enacting a vision for a *kaumātua* housing community. The community agencies were instrumental in creating the vision and integrating *kaumātua* voices early in the development. Community voices are central to applying a culture-centered approach (Dutta, 2007; Oetzel et al., 2017) and ensuring that the project was *kaumātua*-led and centered in *tikanga*. This approach facilitated processes that maintained *kaumātua* independence (*kaumātua mana motuhake*) and enhanced social relationships through a communal space. Furthermore, research argues for housing environments to meet changing needs (Lawton & Simon, 1968; Wahl & Gerstorf, 2020).

Furthermore, specific considerations for “ageing in the right place” (Canham et al., 2021, p. 22) should include social connection, trusting peer and provider relationships, socialization and services tailored to meet residents’ needs, and connections with external community services (Canham et al., 2021). In this study, the culture-centered approach fostered these elements and added an explicit dimension to age-friendly principles of respect and social inclusion, participation, and community support. Furthermore, the orientation of dwellings to a central shared space that supported residents’ care for one another, reflected the culture-centered approach. The project’s approach also helped the proactivity (Wahl & Gerstorf, 2020) of *kaumātua* in co-creating their own life-space concerning the physical, economic, and social environment support.

### Community Codesign

Codesigning the housing community was accomplished through a participatory approach involving shared decision-making and communication (Oetzel et al., 2017). The original visionaries recognized the importance of understanding the local context, housing needs, and legalities around resource consents. In addition, the visionaries recognized their strengths and needs for external expertise by forming partnerships with organizations that shared their vision and could also provide information about funding and finance, resource consents, and construction. This approach is consistent with arguments for greater cross-sector collaboration in creating age-friendly communities (Blakey & Clews, 2020; van Hoof et al., 2021; World Health Organization, 2018) and a systems approach and integrated knowledge translation to design and building processes (Oetzel et al., 2017).

This approach is consistent with community-based participatory research (CBPR) and collaborative partnerships leveraging partners’ strengths throughout a project (Wallerstein et al., 2018). CBPR has long been advocated and utilized to develop sustainable and practical solutions to health and social issues. It helps address power differences based on historical inequities grounded in colonization (Pledger et al., 2019; Wallerstein et al., 2018) and has been used in research to develop supportive housing solutions (Tremblay et al., 2020). Thus, a significant implication of this research is a collaborative and participatory process from inception in developing age-friendly housing communities, especially when working with Indigenous communities.

### Practicalities

The codesign process illustrated the need for research and information around practical decisions such as funding and finance, resource consent, building process, tenancy management, and asset sustainability. These elements are critical for any housing project; however, they are *kaumātua* and culture-centered given the community agencies’ approach. A key aspect of *Māori tikanga* is reverence and care of *kaumātua* (Durie, 2003). The research illustrates the importance of wrap-around services to support residents (e.g., transport, nursing visits, social and cultural events). Another key implication is how codesign and culturally centered processes lead to pragmatic solutions that address *kaumātua* health and social needs. *Kaumātua* face a significant burden of social isolation with negative implications for various health issues (Edwards et al., 2018; Pledger et al., 2019; Wright-St Clair et al., 2017). The *kaumātua papakāinga* addressed these needs by creating shared physical spaces for interaction and dwellings facing this space to encourage that interaction.

Finally, the post-tenancy management contract between Rauawaawa, Te Rūnanga, and the initial funders offers a model for wrap-around service provision, tenancy processes that help those with the greatest need, and property management that maintains the village within a sound financial model. This is an age-friendly management approach in that it supports residents to optimize various dimensions of functioning as they age. It highlights the financial domain of the necessary systematic coordination between service providers, property owners, and residents to maintain and grow an age-friendly housing community. In this respect, the management approach rotates the focus from the recently added individual’s financial situation (Dikken et al., 2020), to include the financial aspects of service provision and coordination.

### Limitations and Conclusions

Despite the strength of culturally centered and in-depth findings, the research has limitations. First is using one site to ground the research findings. The housing village is a long-standing and effective one. Nevertheless, it would be helpful to have additional age-friendly housing communities to support the perspectives advocated in this study. Second is using largely retrospective views. Future research should consider assessing various health and social outcomes pre- and post-build to provide more evidence of the impacts of a new housing community. Such research may include using the Age Friendly Cities and Communities Questionnaire (Dikken et al., 2020), which evaluates older people’s living situations in terms of eight domains of the World Health Age-Friendly Cities model and individual income. Future studies may also explore how current frameworks (van Hoof et al., 2021) capture features of locally developed and culture-centered age-friendly communities.

Accounting for cultural practices in codesigning age-friendly and culture-centered housing for and with Indigenous older adults helps meet their cultural, social, health, and economic needs. It provides advantages compared with alternative models (e.g., residential care or family supported living). In documenting successful codesign processes, this research offers a practical pathway to developing age-friendly housing environments for Māori *kaumātua*, their communities, wider society, and other Indigenous peoples. This study addresses Māori providers’ expressed needs for creative approaches to codesigning culture-centered, age-friendly *kaumātua* communities through housing. As a result, the research can inform the development of Māori and non-Māori policies and practices in the provision of secure, healthy, and affordable homes for *kaumātua* and *whānau*. It can also realize the potential to impact urban and rural Māori organizations by building on and sharing the success factors in this *kaumātua* housing community. Sharing and translating these success factors can help achieve the aspirations of Māori and *kaumātua* for long-term, healthy, and affordable homes to support age-friendly environments.

## Glossary

The first or single use of Māori words (*Kupu Māori*) follows, or is followed by the English translation in the text. The glossary comprises *Kupu Māori* used twice or more.

**Table:** 

Te Reo Māori	English approximation
*Aotearoa*	Māori name of New Zealand
*iwi*	tribe, extended kinship group
*kaitiakitanga*	stewardship
*kaumātua*	Māori elders 55 years and older
*papakāinga*	traditional Māori housing/community complex
*tautokotanga*	support
*te ao Māori*	the Māori world
*te ao mārama*	world of light, being
*te korekore*	world of potential
*te pō*	world of becoming
*tikanga*	Māori cultural practices and protocols
*whakahihikotanga*	inspiration, motivation
*whānau*	closely connected kin group
*whanaungatanga*	connectedness, relationships
